# The Beginnings of a Great Thing

**Published:** 1890-07

**Authors:** 


### THE BEGINNINGS OF A GREAT THING.

The volume of the *Transactions of the American Association of Obstetricians and Gynecologists* for 1889 contains, among a vast mass of valuable matter, a bit of history in the shape of the personal recollections of Dr. Alexander Dunlap, of Springfield, Ohio. A paper on the Refinements of Abdominal Surgery was read by Dr. David Barrow, now of Lexington, Ky., but a former resident student of the Charity Hospital and a graduate of the University of Louisiana. He was followed by Dr. Jos. Hoffman, of Philadelphia, on Some Accidents and Complications Incident and Subsequent to Abdominal and Pelvic Operations.

The discussion on these two papers was lively and instructive. Perhaps the most entertaining remarks were made by Dr. Dunlap. His words were like the echo of a voice of the distant past. It would be difficult to describe more forcibly than Dr. Dunlap the hard experience that ovariotomy had before it was recognized as a lawful surgical procedure. Dr. Dunlap's first ovariotomy was performed in 1843, since which time he has made nearly 400 laparatomies. The president of the association in introducing Dr. Dunlap, said:

> About seventy years ago McDowell made the first ovariotomy. It is almost true to say that the operation for the time being died, after McDowell's time. In the year 1843, in July, the operation referred to by Dr. Price was made by Atlee. Two months later—forty-six years yesterday—the first ovariotomy of the west, after McDowell's, was made by Dr. Alexander Dunlap, whom I now have the pleasure of ntroducing to the association.

Dr. Dunlap disclaimed all right to be called a surgeon when he performed his first ovariotomy. This greatness was forced upon him. When he received his diploma, his authority to cure or do anything else, he felt that he knew but one thing, namely to cure fevers. This valuable knowledge was derived from one of his teachers, Prof. John T. Harrison, who said: "When you have a case of fever salivate it, and as soon as you see signs of ptyalism, you may assure the friends of the patient that their loved one is saved." Dr. Dunlap returned home and began practice with his brother. This same brother was evidently a good observer of human nature, and a man who did not like to be ousted out of anything that he had made for himself, for he said:

Don't disturb me in obstetrics; I have learned enough to know that if you can make a community believe you can do one thing well, they will take all the rest for granted. I advise you to take surgery. * * * If you take up anything as your particular sphere of action, you must study it up and make a success of it, and then you can get credit for all the rest of your ignorance."

This happened in April, 1841, and in the following August a momentous event occurred; he saw his first case of fever! In the encounter, the patient came out second best, but living. Dr. Dunlap said:

It was the most terrific scourge of typhoid fever in Southern Ohio. I had two patients and commenced to salivate them, and in a few days I wished to leave the State. I gave big doses and little doses, but there was no ptyalism, yet on the twenty-first day I thought the two young men to whom I had given that treatment, were getting a little better, and I could say to their friends that their loved ones were safe; but I could not see any salivation, and I could not understand why they were getting better. However, on the twenty-third day I was sure they were better, and on the twenty-fifth day I could smell the calomel. Then, for six weeks I had a fearful time. The patients were getting well, but were cursing me for rotting out their mouths. These cases made me lose faith in my teaching of medicine, and if I ever knew anything about it I would have to learn it myself at the bedside of the patient. Two years after that I got hold of a case of ovarian tumor. I did not know what it was for a while. I had heard that McDowell had operated, and in one little article in an old medical dictionary it was stated that McDowell had done the operation, but it was now condemned and considered to be murderous. I happened to tell this to the patient when I was talking to her; notwithstanding she insisted on my operating. I debated with her until she was almost dead, but she said if I would not do it she would get a common butcher to cut it out. She said: "All I ask is for you to cut me open and let me see what it is, and I will die happy." [Some slanderous critics have affirmed that curiosity is a feminine weakness.]

When urged in this manner to operate, Dr. Dunlap was a graduate of scarcely more than two years' standing. It is well-nigh impossible to appreciate now-a-days the colossal courage that he must have had in order to perform his first ovariotomy. But he finally yielded to his patient's pleadings, and on September 17, 1843, he removed the tumor almost as well, according to him, as he could do it to-day.

That was many years before Listerism entered the lists as the champion of modern surgery. How, then, did Dunlap proceed in order to secure such a good result as he did secure? He says:

> My preparation for the operation was a careful study of the organs of the abdominal cavity, until I had them as plain to my mental vision as they would have been had the cavity been open to my eyes, and I said to myself I would not cut any of them out; but anything else that I found that ought not to be there I would cut out. Fortunately, as soon as I opened the abdomen, I found what I thought ought not to be there. In running my hand around it I burst one big sac. I sponged out the abdomen and turned out the sac, and there was a long pedicle. I could almost have shouted glory! because I knew I could now control the hemorrhage. I finished the operation just as I do to-day. I pierced the pedicle and tied a double knot with silk; I always waxed the silk until I got it thoroughly imbued with wax. The woman lived nearly a month, and was out of all danger from the operation itself. I had been tapping her every ten days, for nearly six months, and drawing out a big wooden bucketful of water each time. The sudden checking of that flow of water from her system brought on a severe diarrhea, with large watery stools. I got that controlled, and she appeared to be doing well, when her kidneys began pouring out a flood of water, and, as I could not stop that, she ran down and died.

Now, when we bear in mind that anesthetics were first practically applied to surgical operations in 1846, we will at once perceive that there was a large element of courage on the part of the patient as well as the operator.

A case of this sort could not be lost to literature; so Dr. Dunlap prepared a report of the case. The fate of that literary effort will strike latter-day surgeons with some surprise:

> I sent a report of the case to Prof. Harrison, and he returned it to me, saying he would not publish such an article, because it would only encourage some other man to do a foolish operation; and I tore it up—what would have been my first contribution to the literature of ovariotomy. Since that day I have done nearly four hundred laparatomies. The last was day before yesterday [*i. e.*, September 16, 1889].

The same age that witnessed the birth and growth of abdominal surgery also witnessed the advent of the steam engine and the telegraph. Progress makes one year as good as ten. The wonderful advances made in a lifetime cause events of fifty years ago to seem almost as belonging to a remote past and Dr. Dunlap, reviewing the changes made in a department of surgery which was scarcely born in his early manhood, may feel like an old-time salt viewing gigantic modern ocean steamers, while his memory carries him back to voyages of four or six months' duration, with their scurvy and other attendant evils.

But a wall grows higher by the stones being piled upon one another; the topmost stones could not reach so high if it were not for the lower stones beneath, neither could an elegant superstructure of a mansion rear aloft if it were not for the plain, unornamental, but indispensable, foundation. Our modern surgery, over which we never tire of crowing, is not a thing living by itself and born of itself; it is merely the imposing capital placed upon the plain but enduring shaft, reared by men like McDowell and Dunlap.

All honor to those pioneers who ushered in the era of fearless, yet saving, surgery.—*New Orleans Med. and Surg. Journal, June.*

